# The Beat

**DOI:** 10.1289/ehp.119-a288b

**Published:** 2011-07-01

**Authors:** Erin E. Dooley

## Gel Tackles Tohoku Waste

CNN reports Japanese officials are using a product called DeconGel to clean up areas contaminated by the March 2011 Tohoku earthquake and tsunami.^1^ The product is applied as a liquid to surfaces contaminated by hazardous chemicals or radiation and then dries into a gel that can be peeled off, taking contaminants with it. DeconGel has been used to clean up polychlorinated biphenyls, mercury, chromium, beryllium, and radioactive materials. Although the product can’t neutralize radioactivity—no product can—its developer claims it can reduce labor and disposal costs.^2^

**Figure:**
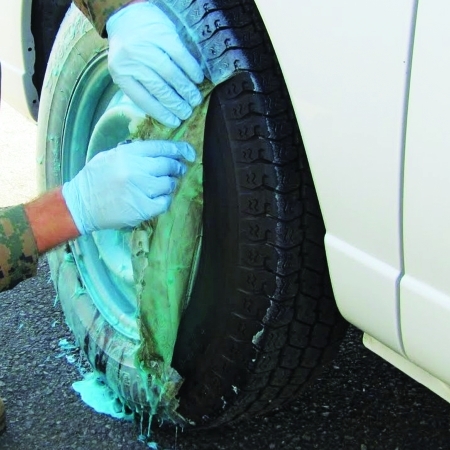
DeconGel being used on a fire truck in Japan. CBI Polymers

## 12th Report on Carcinogens Released

In the 12th *Report on Carcinogens*, released in June 2011, the National Toxicology Program adds two substances to the list of known human carcinogens: formaldehyde (formerly listed as reasonably anticipated to be a human carcinogen) and aristolochic acids (botanical chemicals found in some *Aristolochia*- and *Asarum*-based herbal remedies, which are listed for the first time).^3^ The new report also adds six entries to the list of substances reasonably anticipated to be human carcinogens: capatafol (a fungicide), cobalt–tungsten carbides in powder or hard metal form, certain inhalable glass wool fibers, riddelline (a compound found in *Senecio*-based herbal remedies), and the industrial chemicals *o*-nitrotoluene and styrene.

## IARC Classifies Radiofrequency Electromagnetic Fields

In May 2011 the International Agency for Research on Cancer (IARC) classified radiofrequency electromagnetic fields like those emitted by cell phones as possibly carcinogenic to humans.^4^ The agency based its decision on limited evidence suggesting an increased risk for glioma (a malignant type of brain cancer) and acoustic neuroma (a benign tumor of the nerve connecting the ear to the brain) associated with cell phone use. The group also concluded there is inadequate evidence to draw conclusions for other types of cancers. The number of cell phone users worldwide is currently estimated at more than 5 billion.^5^

## Study to Assess Health Effects of Railyard

In June 2011 California’s South Coast Air Quality Management District announced the launch of a two-year study on the Burlington Northern Santa Fe railyard in San Bernardino, a major transportation hub for the Los Angeles–Long Beach port complex. The study, to be conducted by Loma Linda University, will examine respiratory diseases and cancer among 1,200 children and adults in communities surrounding the railyard. A 2008 California Air Resources Board health risk assessment suggested the San Bernardino facility had the worst health impact on surrounding communities of any railyard in California.^6^

## Elders Volunteer for Fukushima Daiichi Plant Cleanup

Retired engineer Yasuteru Yamada received more than 300 responses when he solicited elderly colleagues to volunteer to help stabilize and clean up the Fukushima Daiichi nuclear plant damaged by the 11 March 2011 Tohoku earthquake and tsunami.^7^ Yamada, a cancer survivor, says elders should lead these efforts because of their accumulated experience and because their advanced age means the adverse effects of radiation exposure will have less time to manifest. Yamada’s Skilled Veterans Corps is expected to receive governmental approval to help at the damaged plant.

**Figure:**
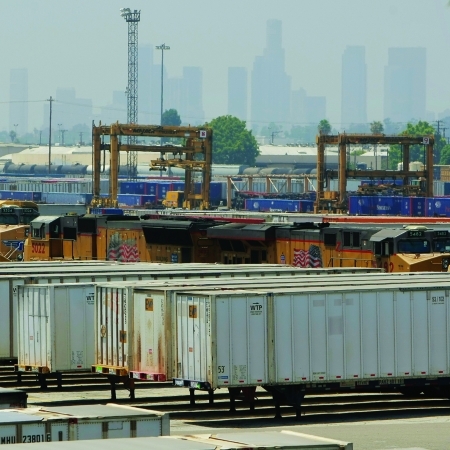
Several projects in California seek to reduce the community health impacts of railyards. AP Photo/Damian Dovarganes
